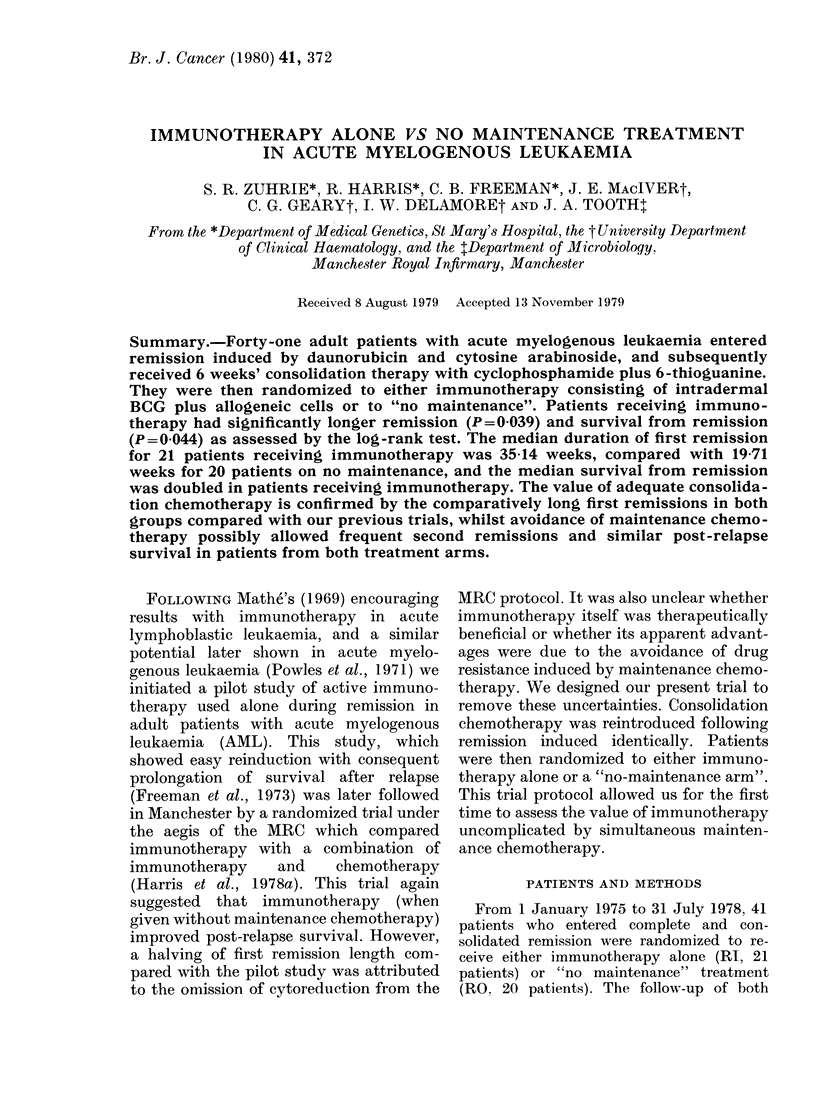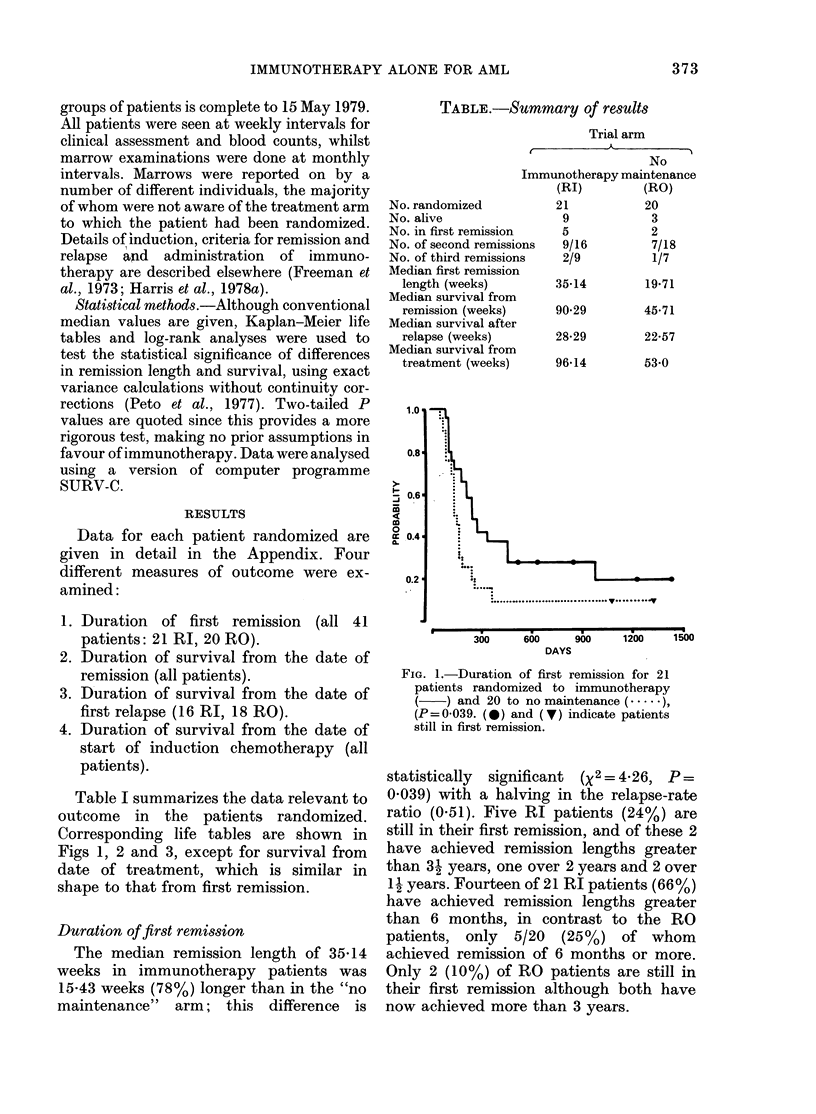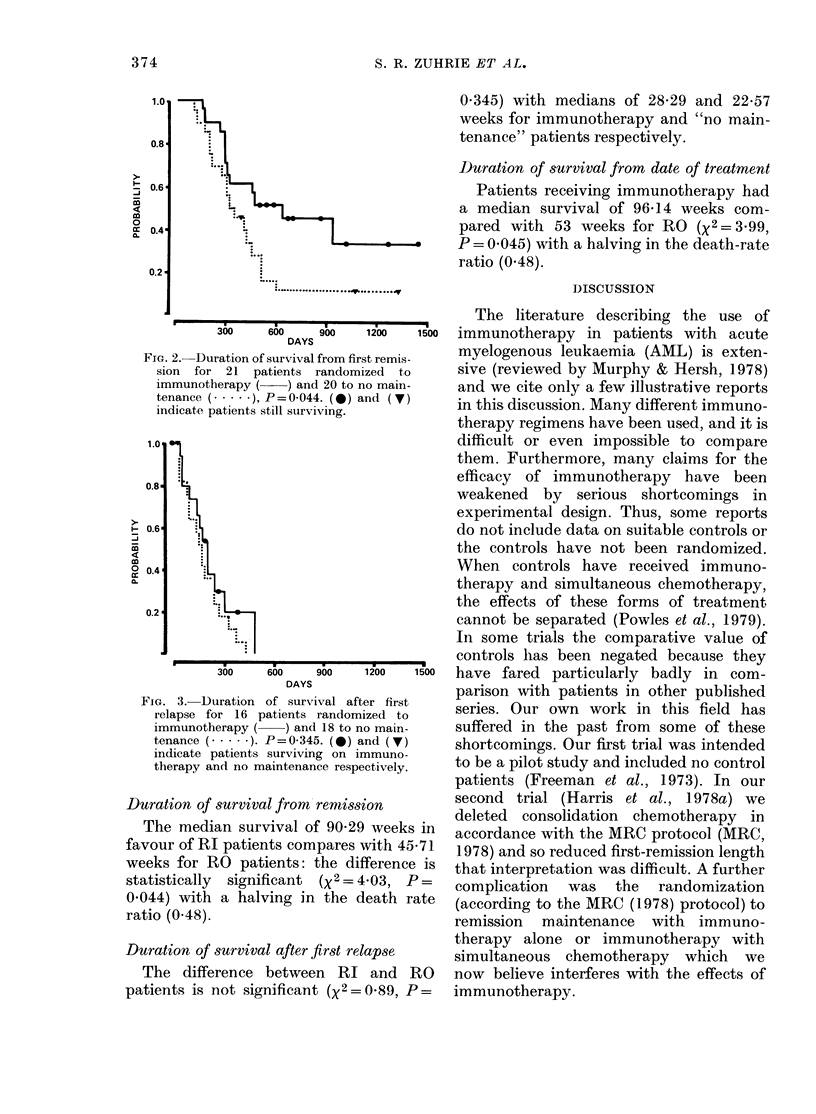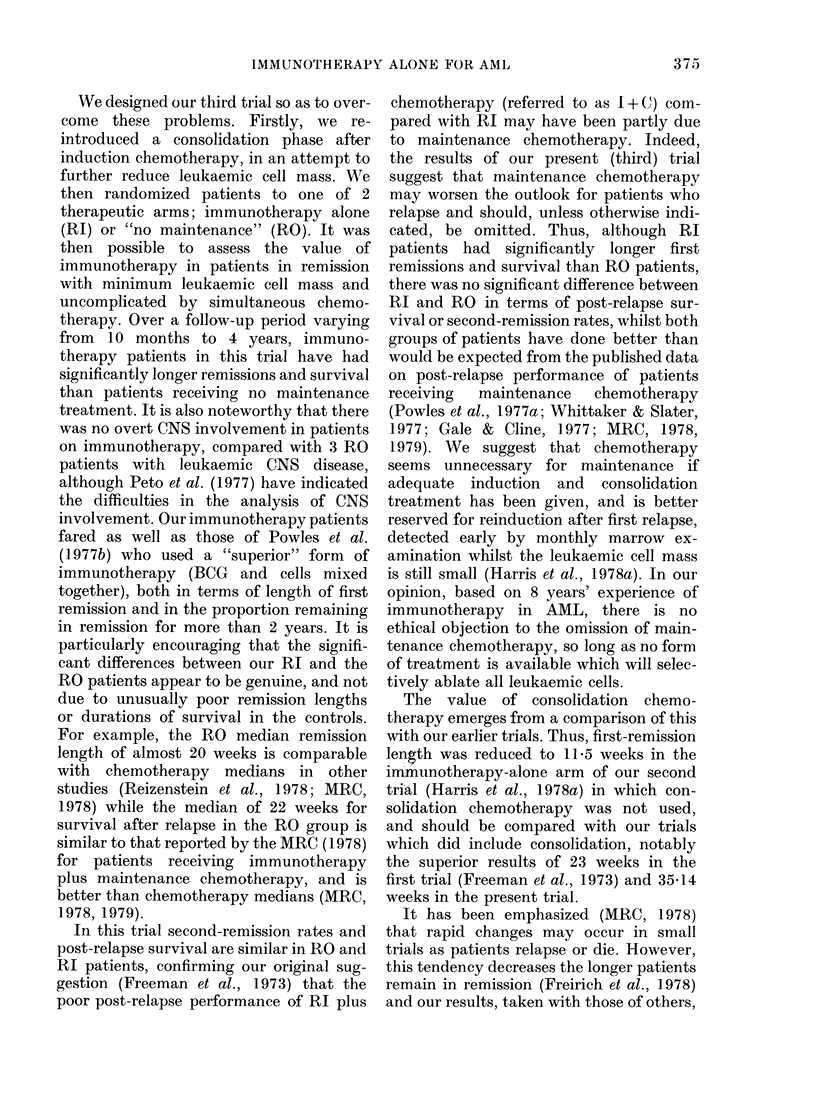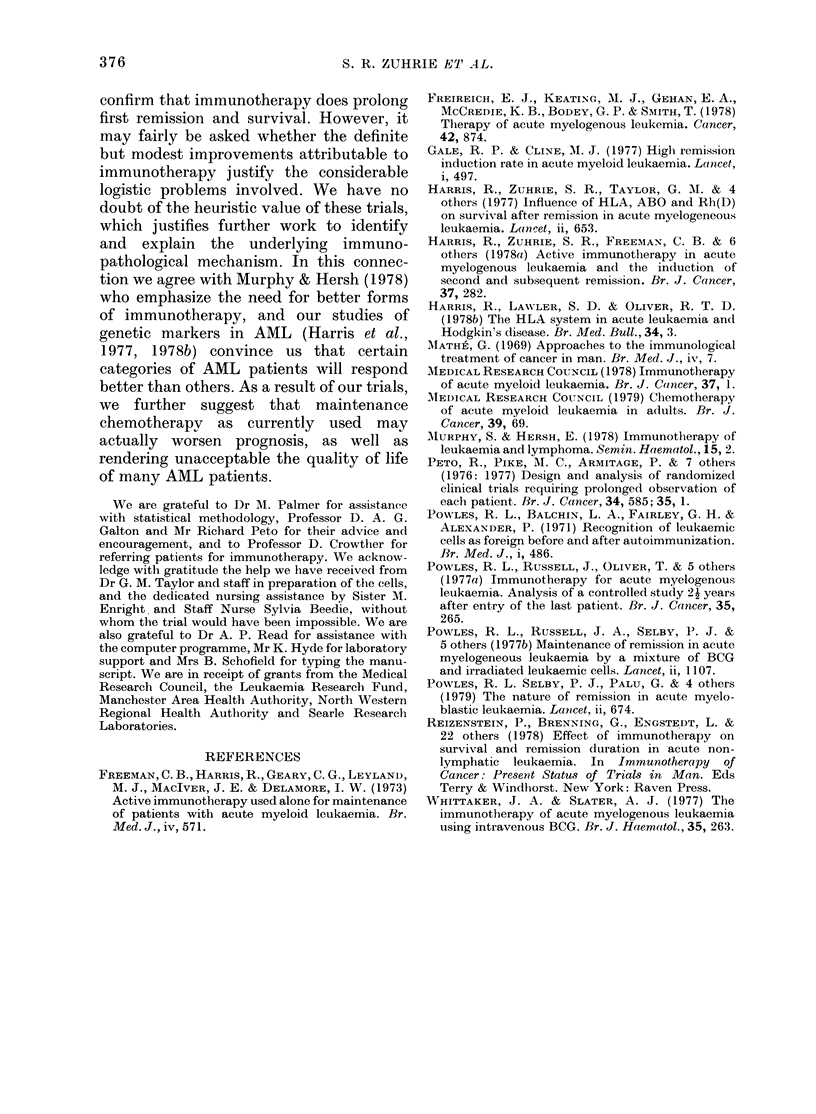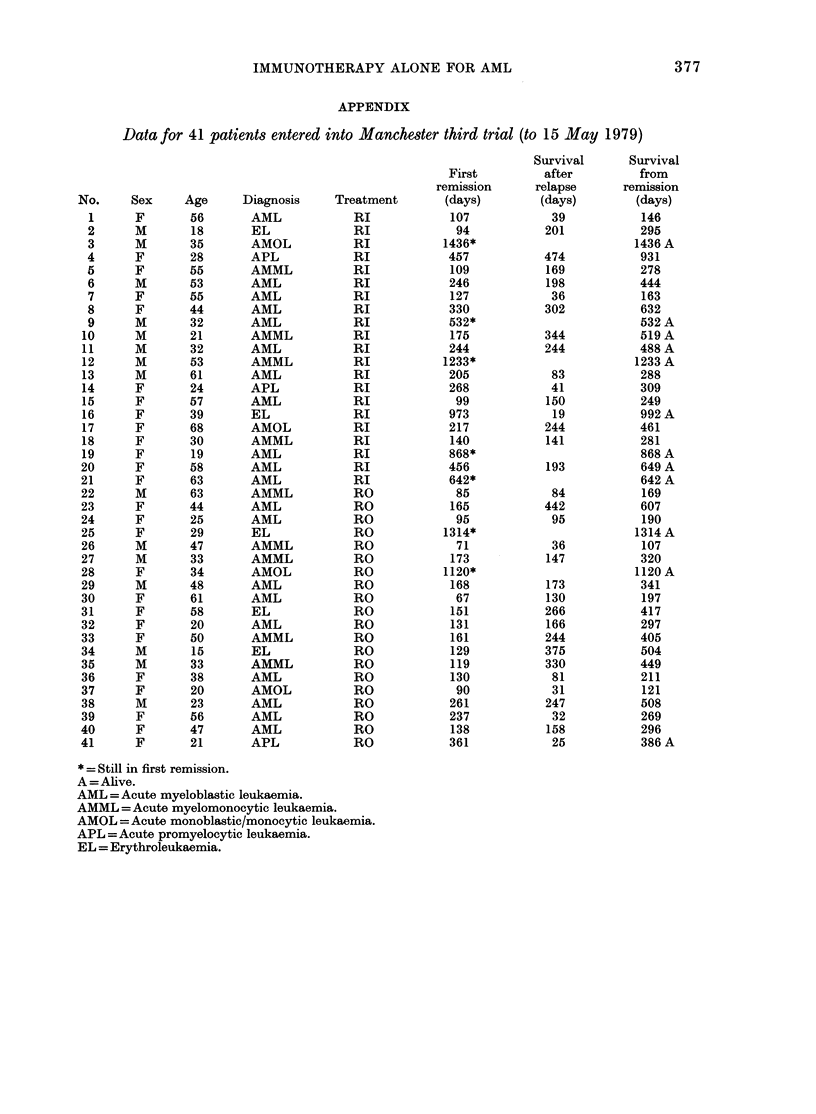# Immunotherapy alone vs no maintenance treatment in acute myelogenous leukaemia.

**DOI:** 10.1038/bjc.1980.60

**Published:** 1980-03

**Authors:** S. R. Zuhrie, R. Harris, C. B. Freeman, J. E. MacIver, C. G. Geary, I. W. Delamore, J. A. Tooth

## Abstract

Forty-one adult patients with acute myelogenous leukaemia entered remission induced by daunorubicin and cytosine arabinoside, and subsequently received 6 weeks' consolidation therapy with cyclophosphamide plus 6-thioguanine. They were then randomized to either immunotherapy consisting of intradermal BCG plus allogeneic cells or to "no maintenance". Patients receiving immunotherapy had significantly longer remission (P = 0.039) and survival from remission (P = 0.044) as assessed by the log-rank test. The median duration of first remission for 21 patients receiving immunotherapy was 35.14 weeks, compared with 19.71 weeks for 20 patients on no maintenance, and the median survival from remission was doubled in patients receiving immunotherapy. The value of adequate consolidation chemotherapy is confirmed by the comparatively long first remissions in both groups compared with our previous trials, whilst avoidance of maintenance chemotherapy possibly allowed frequent second remissions and similar post-relapse survival in patients from both treatment arms.


*Br. J. Cancer* (1980) **41**, 372

### IMMUNOTHERAPY ALONE *VS* NO MAINTENANCE TREATMENT IN ACUTE MYELOGENOUS LEUKAEMIA

S. R. ZUHRIE*, R. HARRIS*, C. B. FREEMAN*, J. E. MacIVER†, C. G. GEARY†, I. W. DELAMORE† AND J. A. TOOTH‡

*From the *Department of Medical Genetics, St Mary's Hospital, the †University Department of Clinical Haematology, and the ‡Department of Microbiology, Manchester Royal Infirmary, Manchester*

Received 8 August 1979 Accepted 13 November 1979

**Summary.—Forty-one adult patients with acute myelogenous leukaemia entered remission induced by daunorubicin and cytosine arabinoside, and subsequently received 6 weeks' consolidation therapy with cyclophosphamide plus 6-thioguanine. They were then randomized to either immunotherapy consisting of intradermal BCG plus allogeneic cells or to "no maintenance". Patients receiving immunotherapy had significantly longer remission ($P=0{\cdot}039$) and survival from remission ($P=0{\cdot}044$) as assessed by the log-rank test. The median duration of first remission for 21 patients receiving immunotherapy was 35·14 weeks, compared with 19·71 weeks for 20 patients on no maintenance, and the median survival from remission was doubled in patients receiving immunotherapy. The value of adequate consolidation chemotherapy is confirmed by the comparatively long first remissions in both groups compared with our previous trials, whilst avoidance of maintenance chemotherapy possibly allowed frequent second remissions and similar post-relapse survival in patients from both treatment arms.**

FOLLOWING Mathé's (1969) encouraging results with immunotherapy in acute lymphoblastic leukaemia, and a similar potential later shown in acute myelogenous leukaemia (Powles *et al.*, 1971) we initiated a pilot study of active immunotherapy used alone during remission in adult patients with acute myelogenous leukaemia (AML). This study, which showed easy reinduction with consequent prolongation of survival after relapse (Freeman *et al.*, 1973) was later followed in Manchester by a randomized trial under the aegis of the MRC which compared immunotherapy with a combination of immunotherapy and chemotherapy (Harris *et al.*, 1978*a*). This trial again suggested that immunotherapy (when given without maintenance chemotherapy) improved post-relapse survival. However, a halving of first remission length compared with the pilot study was attributed to the omission of cytoreduction from the MRC protocol. It was also unclear whether immunotherapy itself was therapeutically beneficial or whether its apparent advantages were due to the avoidance of drug resistance induced by maintenance chemotherapy. We designed our present trial to remove these uncertainties. Consolidation chemotherapy was reintroduced following remission induced identically. Patients were then randomized to either immunotherapy alone or a "no-maintenance arm". This trial protocol allowed us for the first time to assess the value of immunotherapy uncomplicated by simultaneous maintenance chemotherapy.

### PATIENTS AND METHODS

From 1 January 1975 to 31 July 1978, 41 patients who entered complete and consolidated remission were randomized to receive either immunotherapy alone (RI, 21 patients) or "no maintenance" treatment (RO, 20 patients). The follow-up of both groups of patients is complete to 15 May 1979. All patients were seen at weekly intervals for clinical assessment and blood counts, whilst marrow examinations were done at monthly intervals. Marrows were reported on by a number of different individuals, the majority of whom were not aware of the treatment arm to which the patient had been randomized. Details of induction, criteria for remission and relapse and administration of immunotherapy are described elsewhere (Freeman *et al.*, 1973; Harris *et al.*, 1978*a*).

*Statistical methods.*—Although conventional median values are given, Kaplan–Meier life tables and log-rank analyses were used to test the statistical significance of differences in remission length and survival, using exact variance calculations without continuity corrections (Peto *et al.*, 1977). Two-tailed *P* values are quoted since this provides a more rigorous test, making no prior assumptions in favour of immunotherapy. Data were analysed using a version of computer programme SURV-C.

### RESULTS

Data for each patient randomized are given in detail in the Appendix. Four different measures of outcome were examined:

1. Duration of first remission (all 41 patients: 21 RI, 20 RO).
2. Duration of survival from the date of remission (all patients).
3. Duration of survival from the date of first relapse (16 RI, 18 RO).
4. Duration of survival from the date of start of induction chemotherapy (all patients).

Table I summarizes the data relevant to outcome in the patients randomized. Corresponding life tables are shown in Figs 1, 2 and 3, except for survival from date of treatment, which is similar in shape to that from first remission.

### *Duration of first remission*

The median remission length of 35·14 weeks in immunotherapy patients was 15·43 weeks (78%) longer than in the "no maintenance" arm; this difference is statistically significant ($\chi^2 = 4{\cdot}26$, $P = 0{\cdot}039$) with a halving in the relapse-rate ratio (0·51). Five RI patients (24%) are still in their first remission, and of these 2 have achieved remission lengths greater than 3½ years, one over 2 years and 2 over 1½ years. Fourteen of 21 RI patients (66%) have achieved remission lengths greater than 6 months, in contrast to the RO patients, only 5/20 (25%) of whom achieved remission of 6 months or more. Only 2 (10%) of RO patients are still in their first remission although both have now achieved more than 3 years.

TABLE.—*Summary of results*

| | Trial arm: Immunotherapy (RI) | Trial arm: No maintenance (RO) |
|---|---|---|
| No. randomized | 21 | 20 |
| No. alive | 9 | 3 |
| No. in first remission | 5 | 2 |
| No. of second remissions | 9/16 | 7/18 |
| No. of third remissions | 2/9 | 1/7 |
| Median first remission length (weeks) | 35·14 | 19·71 |
| Median survival from remission (weeks) | 90·29 | 45·71 |
| Median survival after relapse (weeks) | 28·29 | 22·57 |
| Median survival from treatment (weeks) | 96·14 | 53·0 |

FIG. 1.—Duration of first remission for 21 patients randomized to immunotherapy (——) and 20 to no maintenance (·····), ($P = 0{\cdot}039$. (●) and (▼) indicate patients still in first remission.

FIG. 2.—Duration of survival from first remission for 21 patients randomized to immunotherapy (——) and 20 to no maintenance (· · · · ·), $P=0{\cdot}044$. (●) and (▼) indicate patients still surviving.

FIG. 3.—Duration of survival after first relapse for 16 patients randomized to immunotherapy (——) and 18 to no maintenance (· · · · ·). $P=0{\cdot}345$. (●) and (▼) indicate patients surviving on immunotherapy and no maintenance respectively.

*Duration of survival from remission*

The median survival of 90·29 weeks in favour of RI patients compares with 45·71 weeks for RO patients: the difference is statistically significant ($\chi^2=4{\cdot}03$, $P=0{\cdot}044$) with a halving in the death rate ratio (0·48).

*Duration of survival after first relapse*

The difference between RI and RO patients is not significant ($\chi^2=0{\cdot}89$, $P=0{\cdot}345$) with medians of 28·29 and 22·57 weeks for immunotherapy and "no maintenance" patients respectively.

*Duration of survival from date of treatment*

Patients receiving immunotherapy had a median survival of 96·14 weeks compared with 53 weeks for RO ($\chi^2=3{\cdot}99$, $P=0{\cdot}045$) with a halving in the death-rate ratio (0·48).

### DISCUSSION

The literature describing the use of immunotherapy in patients with acute myelogenous leukaemia (AML) is extensive (reviewed by Murphy & Hersh, 1978) and we cite only a few illustrative reports in this discussion. Many different immunotherapy regimens have been used, and it is difficult or even impossible to compare them. Furthermore, many claims for the efficacy of immunotherapy have been weakened by serious shortcomings in experimental design. Thus, some reports do not include data on suitable controls or the controls have not been randomized. When controls have received immunotherapy and simultaneous chemotherapy, the effects of these forms of treatment cannot be separated (Powles *et al.*, 1979). In some trials the comparative value of controls has been negated because they have fared particularly badly in comparison with patients in other published series. Our own work in this field has suffered in the past from some of these shortcomings. Our first trial was intended to be a pilot study and included no control patients (Freeman *et al.*, 1973). In our second trial (Harris *et al.*, 1978*a*) we deleted consolidation chemotherapy in accordance with the MRC protocol (MRC, 1978) and so reduced first-remission length that interpretation was difficult. A further complication was the randomization (according to the MRC (1978) protocol) to remission maintenance with immunotherapy alone or immunotherapy with simultaneous chemotherapy which we now believe interferes with the effects of immunotherapy.

We designed our third trial so as to overcome these problems. Firstly, we reintroduced a consolidation phase after induction chemotherapy, in an attempt to further reduce leukaemic cell mass. We then randomized patients to one of 2 therapeutic arms; immunotherapy alone (RI) or "no maintenance" (RO). It was then possible to assess the value of immunotherapy in patients in remission with minimum leukaemic cell mass and uncomplicated by simultaneous chemotherapy. Over a follow-up period varying from 10 months to 4 years, immunotherapy patients in this trial have had significantly longer remissions and survival than patients receiving no maintenance treatment. It is also noteworthy that there was no overt CNS involvement in patients on immunotherapy, compared with 3 RO patients with leukaemic CNS disease, although Peto *et al.* (1977) have indicated the difficulties in the analysis of CNS involvement. Our immunotherapy patients fared as well as those of Powles *et al.* (1977*b*) who used a "superior" form of immunotherapy (BCG and cells mixed together), both in terms of length of first remission and in the proportion remaining in remission for more than 2 years. It is particularly encouraging that the significant differences between our RI and the RO patients appear to be genuine, and not due to unusually poor remission lengths or durations of survival in the controls. For example, the RO median remission length of almost 20 weeks is comparable with chemotherapy medians in other studies (Reizenstein *et al.*, 1978; MRC, 1978) while the median of 22 weeks for survival after relapse in the RO group is similar to that reported by the MRC (1978) for patients receiving immunotherapy plus maintenance chemotherapy, and is better than chemotherapy medians (MRC, 1978, 1979).

In this trial second-remission rates and post-relapse survival are similar in RO and RI patients, confirming our original suggestion (Freeman *et al.*, 1973) that the poor post-relapse performance of RI plus chemotherapy (referred to as I+C) compared with RI may have been partly due to maintenance chemotherapy. Indeed, the results of our present (third) trial suggest that maintenance chemotherapy may worsen the outlook for patients who relapse and should, unless otherwise indicated, be omitted. Thus, although RI patients had significantly longer first remissions and survival than RO patients, there was no significant difference between RI and RO in terms of post-relapse survival or second-remission rates, whilst both groups of patients have done better than would be expected from the published data on post-relapse performance of patients receiving maintenance chemotherapy (Powles *et al.*, 1977*a*; Whittaker & Slater, 1977; Gale & Cline, 1977; MRC, 1978, 1979). We suggest that chemotherapy seems unnecessary for maintenance if adequate induction and consolidation treatment has been given, and is better reserved for reinduction after first relapse, detected early by monthly marrow examination whilst the leukaemic cell mass is still small (Harris *et al.*, 1978*a*). In our opinion, based on 8 years' experience of immunotherapy in AML, there is no ethical objection to the omission of maintenance chemotherapy, so long as no form of treatment is available which will selectively ablate all leukaemic cells.

The value of consolidation chemotherapy emerges from a comparison of this with our earlier trials. Thus, first-remission length was reduced to 11·5 weeks in the immunotherapy-alone arm of our second trial (Harris *et al.*, 1978*a*) in which consolidation chemotherapy was not used, and should be compared with our trials which did include consolidation, notably the superior results of 23 weeks in the first trial (Freeman *et al.*, 1973) and 35·14 weeks in the present trial.

It has been emphasized (MRC, 1978) that rapid changes may occur in small trials as patients relapse or die. However, this tendency decreases the longer patients remain in remission (Freirich *et al.*, 1978) and our results, taken with those of others, confirm that immunotherapy does prolong first remission and survival. However, it may fairly be asked whether the definite but modest improvements attributable to immunotherapy justify the considerable logistic problems involved. We have no doubt of the heuristic value of these trials, which justifies further work to identify and explain the underlying immunopathological mechanism. In this connection we agree with Murphy & Hersh (1978) who emphasize the need for better forms of immunotherapy, and our studies of genetic markers in AML (Harris *et al.*, 1977, 1978*b*) convince us that certain categories of AML patients will respond better than others. As a result of our trials, we further suggest that maintenance chemotherapy as currently used may actually worsen prognosis, as well as rendering unacceptable the quality of life of many AML patients.

We are grateful to Dr M. Palmer for assistance with statistical methodology, Professor D. A. G. Galton and Mr Richard Peto for their advice and encouragement, and to Professor D. Crowther for referring patients for immunotherapy. We acknowledge with gratitude the help we have received from Dr G. M. Taylor and staff in preparation of the cells, and the dedicated nursing assistance by Sister M. Enright and Staff Nurse Sylvia Beedie, without whom the trial would have been impossible. We are also grateful to Dr A. P. Read for assistance with the computer programme, Mr K. Hyde for laboratory support and Mrs B. Schofield for typing the manuscript. We are in receipt of grants from the Medical Research Council, the Leukaemia Research Fund, Manchester Area Health Authority, North Western Regional Health Authority and Searle Research Laboratories.

### REFERENCES

FREEMAN, C. B., HARRIS, R., GEARY, C. G., LEYLAND, M. J., MACIVER, J. E. & DELAMORE, I. W. (1973) Active immunotherapy used alone for maintenance of patients with acute myeloid leukaemia. *Br. Med. J.*, iv, 571.

FREIREICH, E. J., KEATING, M. J., GEHAN, E. A., MCCREDIE, K. B., BODEY, G. P. & SMITH, T. (1978) Therapy of acute myelogenous leukemia. *Cancer*, **42**, 874.

GALE, R. P. & CLINE, M. J. (1977) High remission induction rate in acute myeloid leukaemia. *Lancet*, i, 497.

HARRIS, R., ZUHRIE, S. R., TAYLOR, G. M. & 4 others (1977) Influence of HLA, ABO and Rh(D) on survival after remission in acute myelogeneous leukaemia. *Lancet*, ii, 653.

HARRIS, R., ZUHRIE, S. R., FREEMAN, C. B. & 6 others (1978*a*) Active immunotherapy in acute myelogenous leukaemia and the induction of second and subsequent remission. *Br. J. Cancer*, **37**, 282.

HARRIS, R., LAWLER, S. D. & OLIVER, R. T. D. (1978*b*) The HLA system in acute leukaemia and Hodgkin's disease. *Br. Med. Bull.*, **34**, 3.

MATHÉ, G. (1969) Approaches to the immunological treatment of cancer in man. *Br. Med. J.*, iv, 7.

MEDICAL RESEARCH COUNCIL (1978) Immunotherapy of acute myeloid leukaemia. *Br. J. Cancer*, **37**, 1.

MEDICAL RESEARCH COUNCIL (1979) Chemotherapy of acute myeloid leukaemia in adults. *Br. J. Cancer*, **39**, 69.

MURPHY, S. & HERSH, E. (1978) Immunotherapy of leukaemia and lymphoma. *Semin. Haematol.*, **15**, 2.

PETO, R., PIKE, M. C., ARMITAGE, P. & 7 others (1976: 1977) Design and analysis of randomized clinical trials requiring prolonged observation of each patient. *Br. J. Cancer*, **34**, 585; **35**, 1.

POWLES, R. L., BALCHIN, L. A., FAIRLEY, G. H. & ALEXANDER, P. (1971) Recognition of leukaemic cells as foreign before and after autoimmunization. *Br. Med. J.*, i, 486.

POWLES, R. L., RUSSELL, J., OLIVER, T. & 5 others (1977*a*) Immunotherapy for acute myelogenous leukaemia. Analysis of a controlled study 2½ years after entry of the last patient. *Br. J. Cancer*, **35**, 265.

POWLES, R. L., RUSSELL, J. A., SELBY, P. J. & 5 others (1977*b*) Maintenance of remission in acute myelogeneous leukaemia by a mixture of BCG and irradiated leukaemic cells. *Lancet*, ii, 1107.

POWLES, R. L. SELBY, P. J., PALU, G. & 4 others (1979) The nature of remission in acute myeloblastic leukaemia. *Lancet*, ii, 674.

REIZENSTEIN, P., BRENNING, G., ENGSTEDT, L. & 22 others (1978) Effect of immunotherapy on survival and remission duration in acute nonlymphatic leukaemia. In *Immunotherapy of Cancer: Present Status of Trials in Man.* Eds Terry & Windhorst. New York: Raven Press.

WHITTAKER, J. A. & SLATER, A. J. (1977) The immunotherapy of acute myelogenous leukaemia using intravenous BCG. *Br. J. Haematol.*, **35**, 263.

### APPENDIX

*Data for* 41 *patients entered into Manchester third trial* (*to* 15 *May* 1979)

| No. | Sex | Age | Diagnosis | Treatment | First remission (days) | Survival after relapse (days) | Survival from remission (days) |
|---|---|---|---|---|---|---|---|
| 1 | F | 56 | AML | RI | 107 | 39 | 146 |
| 2 | M | 18 | EL | RI | 94 | 201 | 295 |
| 3 | M | 35 | AMOL | RI | 1436* | | 1436 A |
| 4 | F | 28 | APL | RI | 457 | 474 | 931 |
| 5 | F | 55 | AMML | RI | 109 | 169 | 278 |
| 6 | M | 53 | AML | RI | 246 | 198 | 444 |
| 7 | F | 55 | AML | RI | 127 | 36 | 163 |
| 8 | F | 44 | AML | RI | 330 | 302 | 632 |
| 9 | M | 32 | AML | RI | 532* | | 532 A |
| 10 | M | 21 | AMML | RI | 175 | 344 | 519 A |
| 11 | M | 32 | AML | RI | 244 | 244 | 488 A |
| 12 | M | 53 | AMML | RI | 1233* | | 1233 A |
| 13 | M | 61 | AML | RI | 205 | 83 | 288 |
| 14 | F | 24 | APL | RI | 268 | 41 | 309 |
| 15 | F | 57 | AML | RI | 99 | 150 | 249 |
| 16 | F | 39 | EL | RI | 973 | 19 | 992 A |
| 17 | F | 68 | AMOL | RI | 217 | 244 | 461 |
| 18 | F | 30 | AMML | RI | 140 | 141 | 281 |
| 19 | F | 19 | AML | RI | 868* | | 868 A |
| 20 | F | 58 | AML | RI | 456 | 193 | 649 A |
| 21 | F | 63 | AML | RI | 642* | | 642 A |
| 22 | M | 63 | AMML | RO | 85 | 84 | 169 |
| 23 | F | 44 | AML | RO | 165 | 442 | 607 |
| 24 | F | 25 | AML | RO | 95 | 95 | 190 |
| 25 | F | 29 | EL | RO | 1314* | | 1314 A |
| 26 | M | 47 | AMML | RO | 71 | 36 | 107 |
| 27 | M | 33 | AMML | RO | 173 | 147 | 320 |
| 28 | F | 34 | AMOL | RO | 1120* | | 1120 A |
| 29 | M | 48 | AML | RO | 168 | 173 | 341 |
| 30 | F | 61 | AML | RO | 67 | 130 | 197 |
| 31 | F | 58 | EL | RO | 151 | 266 | 417 |
| 32 | F | 20 | AML | RO | 131 | 166 | 297 |
| 33 | F | 50 | AMML | RO | 161 | 244 | 405 |
| 34 | M | 15 | EL | RO | 129 | 375 | 504 |
| 35 | M | 33 | AMML | RO | 119 | 330 | 449 |
| 36 | F | 38 | AML | RO | 130 | 81 | 211 |
| 37 | F | 20 | AMOL | RO | 90 | 31 | 121 |
| 38 | M | 23 | AML | RO | 261 | 247 | 508 |
| 39 | F | 56 | AML | RO | 237 | 32 | 269 |
| 40 | F | 47 | AML | RO | 138 | 158 | 296 |
| 41 | F | 21 | APL | RO | 361 | 25 | 386 A |

* = Still in first remission.
A = Alive.
AML = Acute myeloblastic leukaemia.
AMML = Acute myelomonocytic leukaemia.
AMOL = Acute monoblastic/monocytic leukaemia.
APL = Acute promyelocytic leukaemia.
EL = Erythroleukaemia.